# A structural study of TatD from *Staphylococcus aureus* elucidates a putative DNA-binding mode of a Mg^2+^-dependent nuclease

**DOI:** 10.1107/S2052252520003917

**Published:** 2020-04-17

**Authors:** Kyu-Yeon Lee, Seung-Ho Cheon, Dong-Gyun Kim, Sang Jae Lee, Bong-Jin Lee

**Affiliations:** a Research Institute of Pharmaceutical Sciences, College of Pharmacy, Seoul National University, Seoul 08826, Republic of Korea; b PAL-XFEL, Pohang Accelerator Laboratory, POSTECH, Pohang, Gyeongbuk 37673, Republic of Korea

**Keywords:** TatD, *Staphylococcus aureus*, metal-dependent nuclease, DNA-binding protein, structure determination, protein structure, refinement, X-ray crystallography, enzyme mechanisms

## Abstract

TatD has been thoroughly investigated as a DNA-repair enzyme and an apoptotic nuclease, and still-unknown TatD-related DNases are considered to play crucial cellular roles. However, studies of TatD from Gram-positive bacteria have been hindered by an absence of atomic detail and the resulting inability to determine function from structure. In this study, an X-ray crystal structure of SAV0491, which is the TatD enzyme from the Gram-positive bacterium *Staphylococcus aureus* (*Sa*TatD), is reported at a high resolution of 1.85 Å with a detailed atomic description. Although *Sa*TatD has the common TIM-barrel fold shared by most TatD-related homologs, and PDB entry 2gzx shares 100% sequence identity with SAV0491, the crystal structure of *Sa*TatD revealed a unique binding mode of two phosphates interacting with two Ni^2+^ ions. Through a functional study, it was verified that *Sa*TatD has Mg^2+^-dependent nuclease activity as a DNase and an RNase. In addition, structural comparison with TatD homologs and the identification of key residues contributing to the binding mode of Ni^2+^ ions and phosphates allowed mutational studies to be performed that revealed the catalytic mechanism of *Sa*TatD. Among the key residues composing the active site, the acidic residues Glu92 and Glu202 had a critical impact on catalysis by *Sa*TatD. Furthermore, based on the binding mode of the two phosphates and structural insights, a putative DNA-binding mode of *Sa*TatD was proposed using *in silico* docking. Overall, these findings may serve as a good basis for understanding the relationship between the structure and function of TatD proteins from Gram-positive bacteria and may provide critical insights into the DNA-binding mode of *Sa*TatD.

## Abstract 2

The crystal structure of SAV0491, a TatD-related DNase from the Gram-positive bacterium *Staphylococcus aureus*, was determined at 1.85 Å resolution, providing functional and structural insights into a putative DNA-binding mode of SAV0491.

## 1. Introduction   

The twin-arginine translocation (Tat) pathway exists in most prokaryotes. The *tat* operon encodes Tat proteins that play an important role in the transportation of completely folded proteins across the bacterial cytoplasm to the periplasm (Sargent *et al.*, 1998 ▸; Hu *et al.*, 2010 ▸). The Tat system in most Gram-positive bacteria consists of three components: TatA, TatC and TatD proteins (Chen, Li *et al.*, 2014 ▸; Freudl, 2013 ▸; Biswas *et al.*, 2009 ▸). TatA and TatC are membrane-bound proteins and are essential for the export of folded proteins with twin-arginine signal peptides, whereas the TatD protein is located in the cytoplasm with Mg^2+^-dependent DNase activity and expression of TatD is not essential for protein transportation in the Tat pathway (Chen, Li *et al.*, 2014 ▸; Freudl, 2013 ▸). However, although not required for the Tat export system, TatD is a crucial element of a Tat-system-linked quality-control system that degrades misfolded or mutated proteins and then inhibits the export of such proteins (Matos *et al.*, 2009 ▸). In addition, TatD is involved in DNA cleavage during apoptosis in *Saccharomyces cerevisiae* (Qiu *et al.*, 2005 ▸) and processes damaged DNA during DNA repair (Chen, Li *et al.*, 2014 ▸).

TatD is a protein that is widely presented and conserved in various species, both prokaryotes and eukaryotes (Wang *et al.*, 2018 ▸), and it is also involved in the DNA-repair system, in apoptotic cell death and in cleaving neutrophil extracellular traps (Chang *et al.*, 2016 ▸; Chen, Shen *et al.*, 2014 ▸; Gannavaram & Debrabant, 2012 ▸; Qiu *et al.*, 2005 ▸). A variety of genes encoding TatD-like proteins, including a single conserved TatD domain consisting of approximately 250 amino acids, have been reported (Chen, Li *et al.*, 2014 ▸). However, while these proteins have been widely investigated in Gram-negative bacteria (Chen, Li *et al.*, 2014 ▸; Matos *et al.*, 2009 ▸; Chang *et al.*, 2016 ▸), represented by *Escherichia coli*, little has been reported regarding the structural and functional properties of TatD-like proteins in Gram-positive bacteria. SAV0491, a putative deoxyribonuclease from the Gram-positive bacterium *Staphylococcus aureus* (Mu50 strain), shares sequence identities of 30% and 34% with the TatD proteins from *E. coli* and *Homo sapiens*, respectively. Although the crystal structure (PDB entry 2gzx; Northeast Structural Genomics Consortium, unpublished work) of MW0446 from *S. aureus* (MW2 strain) shares 100% sequence identity with *Sa*TatD, we focused on SAV0491 to thoroughly investigate the structural and functional features of TatD-like DNases from Gram-positive bacteria.

This study aimed to elucidate the crystal structure of SAV0491, a TatD-like DNase from *Staphylococcus aureus* (*Sa*TatD). We described the crystal structure of *Sa*TatD at the atomic level and identified key information regarding the active site of the enzyme, which coordinates two nickel ions (Ni^2+^) and two phosphate ions. A metal-dependent nuclease activity test revealed that *Sa*TatD critically employs Mg^2+^ ions for its catalytic reaction. In addition, based on the structural insight into the active site, we found eight residues, His6, His8, His63, His128, His153, Glu92, Glu202 and Asp204, which contribute to metal binding and DNA hydrolysis, and identified the DNase activities of their alanine mutants in an investigation of catalytically important residues. Furthermore, *in silico* docking based on two phosphate molecules located in the active site developed further understanding of a putative DNA-binding mode of *Sa*TatD. Taken together, our findings in this study provide considerable insights into the structure of *Sa*TatD from *S. aureus* and reveal novelties in its active site and enzymatic mechanism.

## 2. Materials and methods   

### 2.1. Gene cloning, protein expression and purification of wild-type *Sa*TatD   

The gene encoding SAV0491 from *S. aureus* was amplified by polymerase chain reaction (PCR) using *S. aureus* (Mu50 strain, ATCC 700699) genomic DNA (Kuroda *et al.*, 2001 ▸) as a template. The PCR products and pET-28a vector (Merck Millipore, Germany) were digested with both NdeI and XhoI (NEB, UK) and ligated. After verifying the DNA sequence, the recombinant protein was overexpressed in *E. coli* Rosetta 2(DE3)pLysS cells (Sigma–Aldrich, USA) and grown at 37°C in Luria–Bertani (LB) broth and M9 minimal broth to obtain native and selenomethionine (SeMet)-derived proteins, respectively. When the cells reached an OD_600_ of 0.5, 0.5 m*M* isopropyl β-d-1-thiogalactopyranoside (IPTG) was added to induce protein expression. To obtain SeMet-derived protein, 50 mg l^−1^ l-SeMet was added to the culture medium 30 min before IPTG induction. The cells were then transferred to a 15°C incubator and grown for an additional 24 h. The cells were harvested by centrifugation at 4300*g* for 10 min. The cell pellet was resuspended in lysis buffer [50 m*M* Tris, 500 m*M* NaCl pH 8.0, 10%(*v*/*v*) glycerol] and then sonicated at 4°C. The lysate was centrifuged at 18 000*g* for 60 min at 4°C, and the supernatant was loaded onto a HiTrap Chelating HP column (GE Healthcare) that had been pre-equilibrated with lysis buffer. The column was washed with a 30-fold excess volume of lysis buffer containing 20 m*M* imidazole. The protein eluted at an imidazole concentration in the range 100–250 m*M*. The collected fractions were concentrated using an Amicon Ultra-15 Centrifugal Filter Unit with a 10K molecular-weight cutoff (Millipore, USA). The *Sa*TatD protein was further purified by gel filtration on a HiLoad 16/600 Superdex 200 prep-grade column (GE Healthcare) that had been pre-equilibrated with buffer *A* (20 m*M* Tris, 150 m*M* NaCl pH 8.0).

### 2.2. Construction, protein expression and purification of *Sa*TatD mutants   

The wild-type plasmid harboring the *SAV0491* gene was used in site-directed mutagenesis (SDM) as a template for PCR with an EZchange Site-directed Mutagenesis Kit (Enzynomics, Republic of Korea) according to the manufacturer’s instructions. The mutagenic primers designed for the construction of the H6A, H8A, H63A, H128A, H153A, E92A, E202A and D204A mutants are listed in Supplementary Table S1. All mutant sequences were verified by a commercial sequencing service. The *Sa*TatD mutant proteins were overexpressed, purified and quantitated as described above for the wild type.

### 2.3. Crystallization and X-ray data collection   

Crystallization was performed with manufactured screening kits using the sitting-drop vapor-diffusion method. Native SAV0491 crystals were grown at 20°C by mixing 1.1 µl protein solution (9 mg ml^−1^ in buffer *A*) and 0.7 µl reservoir solution (2.4 *M* sodium malonate pH 7.0). SeMet-derived crystals were grown under the same conditions as native crystals by mixing equal volumes (1.0 µl) of SeMet-derived protein solution (9 mg ml^−1^ in buffer *A*) and reservoir solution. Before data collection, 20%(*v*/*v*) glycerol was added to each crystallization solution and the mixture was used to protect the crystals during flash-cooling by liquid nitrogen. Single-wavelength X-ray diffraction data for SAV0491 were collected at λ = 0.97930 using an ADSC Q315r CCD detector on beamline PAL-5C (SBII) at the Pohang Light Source, Republic of Korea. All data sets were processed and scaled with the *HKL-2000* software package (Otwinowski & Minor, 1997 ▸).

### 2.4. Structure determination, refinement and analysis   

The crystal structure of *Sa*TatD was determined by single-wavelength anomalous dispersion (SAD) with *phenix.autosol* in the *Phenix* software suite (Liebschner *et al.*, 2019 ▸). The initial structure was built using *phenix.autobuild* and further refined using *phenix.refine* and *REFMAC* (Murshudov *et al.*, 2011 ▸). The models were manually constructed and ligands were added using *Coot* (Emsley *et al.*, 2010 ▸). 5% of the data were randomly set aside as test data for the calculation of *R*_free_ (Brünger, 1992 ▸). The structural deviations were calculated using the secondary-structure matching (SSM) superpose option in *Coot*. The solvent-accessible surface areas were calculated using *PISA* (Krissinel & Henrick, 2007 ▸) and the protein–ligand interactions were calculated using *LigPlot* (Wallace *et al.*, 1995 ▸).

### 2.5. Inductively coupled plasma mass spectrometry   

Inductively coupled plasma mass spectrometry (ICP-MS) was conducted to identify which metal ions were present in the crystal structure of *Sa*TatD. A high concentration of the purified *Sa*TatD protein (14 mg ml^−1^) and an abundance of *Sa*TatD crystals dissolved in water were prepared and analyzed for the presence of Mg^2+^, Ca^2+^, Mn^2+^, Ni^2+^ and Zn^2+^. Before measurement, the samples were denatured in nitric acid using a microwave at 1200–1800 W. ICP-MS (7900 ICP-MS, Agilent, USA) and argon plasma (6000–8000 K) were used for detection. The radio-frequency power and matching were 1550 W and 1.72 V, respectively. All data are shown using the average of three independent experiments.

### 2.6. Analytical gel filtration   

The purified *Sa*TatD proteins (at a concentration of 0.5 or 20.0 mg ml^−1^) were subjected to analytical gel-filtration chromatography on a Superdex 200 (10/300 GL) column that had been pre-equilibrated with buffer *A* at a constant flow rate of 0.5 ml min^−1^. A standard curve was obtained using gel-filtration standards (Bio-Rad, catalog No. 1511901) including thyroglobulin (650 kDa), γ-globulin (158 kDa), ovalbumin (44 kDa) and myoglobin (17 kDa). The experimental molecular weights of TatD were calculated using *Microsoft Excel*.

### 2.7. *In vitro* deoxyribonuclease (DNase) assay   

*Sa*TatD protein was prepared and incubated with 4 m*M* EDTA for an hour to remove nonspecifically bound metals and was then desalted using a HiPrep 26/10 Desalting column (GE Healthcare) pre-equilibrated with buffer *A*. Genomic DNA of *Sa*TatD (771 bp) was amplified by PCR using a plasmid harboring the *SAV0491* gene as a template and was purified using a PCR purification kit (Favorgen). A DNase assay was performed in the presence of 2 m*M* bivalent magnesium (MgCl_2_), manganese (MnCl_2_) and calcium (CaCl_2_) and 0.2 m*M* zinc ions (ZnCl_2_) with 200 n*M* genomic DNA of *Sa*TatD as a substrate. Reactions were initiated by the addition of 10 µ*M* protein and were performed for 1 h at 37°C. A protein or metal concentration-dependent DNase assay was performed in the presence of 2 m*M* Mg^2+^ with 0–50 µ*M* *Sa*TatD protein or in the presence of 4 µ*M* *Sa*TatD protein with 0.1–50 m*M* Mg^2+^ using 100 n*M* *Sa*TatD genomic DNA. For the identification of metals in the *Sa*TatD structure, *Sa*TatD protein prepared for crystallization was used in the DNase assay with the same procedure as described above for 24 h at 37°C. A DNase assay of *Sa*TatD mutants was carried out with each mutant protein at 4 µ*M*, 2 m*M* Mg^2+^ and 200 n*M* *Sa*TatD genomic DNA as described above. All reaction mixtures were incubated for 1 h at 37°C and electrophoresed on a 1.5% agarose gel in 0.5× Tris–borate–EDTA (TBE) buffer. Gel images were visualized using a WSE-5200 Printgraph 2M (ATTO).

### 2.8. *In vitro* ribonuclease (RNase) assay   

RNase activity was measured by a fluorescence-quenching assay using an RNase Alert kit (IDT). In this assay system, a fluorophore is covalently linked to one end of a strand of RNA substrate and quenched by a quencher group at the other end. When ribonuclease is added to the RNA substrate containing a fluorophore–quencher pair, digestion of the RNA results in the separation of fluorophore and quencher and fluorescent emission (Em) at 520 nm upon excitation (Ex) at 490 nm by a fluorometer. The assay was performed with 0.2 nmol ml^−1^ RNase substrate, 1× reaction buffer and 4 m*M* EDTA or 2 m*M* MgCl_2_, MnCl_2_, CaCl_2_ or 0.2 m*M* ZnCl_2_ in 383-well black and clear bottom plates. After 10 µ*M* *Sa*TatD protein was added to each well other than the control-group wells, fluorescence values were measured every minute for 1 h using a SpectraMax M5 microplate reader (Molecular Devices) at 37°C.

### 2.9. *In silico* *Sa*TatD–DNA docking   

To further understand the DNA-binding mode of *Sa*TatD, an *in silico* molecular-docking study was performed using the high ambiguity driven protein–protein docking (*HADDOCK*) algorithm (Dominguez *et al.*, 2003 ▸). The coordinates for the *Sa*TatD protein were taken from the determined crystal structure but with the ligands removed, and the coordinates for A-DNA molecules spanning 3 (5′-GCT-3′) or 18 base pairs (5′-GCTGCTGCTGCTGCTGCT-3′) were modeled using 3*D-DART* (van Dijk & Bonvin, 2009 ▸). The residues His8, His63, Glu92, His128, Ser154 and Asp204, which were involved in the binding of phosphates and G1–T3 of the DNA, were defined as ‘active residues’ and were required to have an interface contact of ambiguous distance. Passive residues were defined automatically as residues around active residues.

### 2.10. Data availability   

Coordinates and structural factors have been deposited in the Research Collaboratory for Structural Bioinformatics (RCSB) Protein Data Bank (PDB) under the accession code 6l25 for *Sa*TatD, the TatD-like DNase from *S. aureus*.

## 3. Results   

### 3.1. Crystal structure of *Sa*TatD   

**Figure 1:**
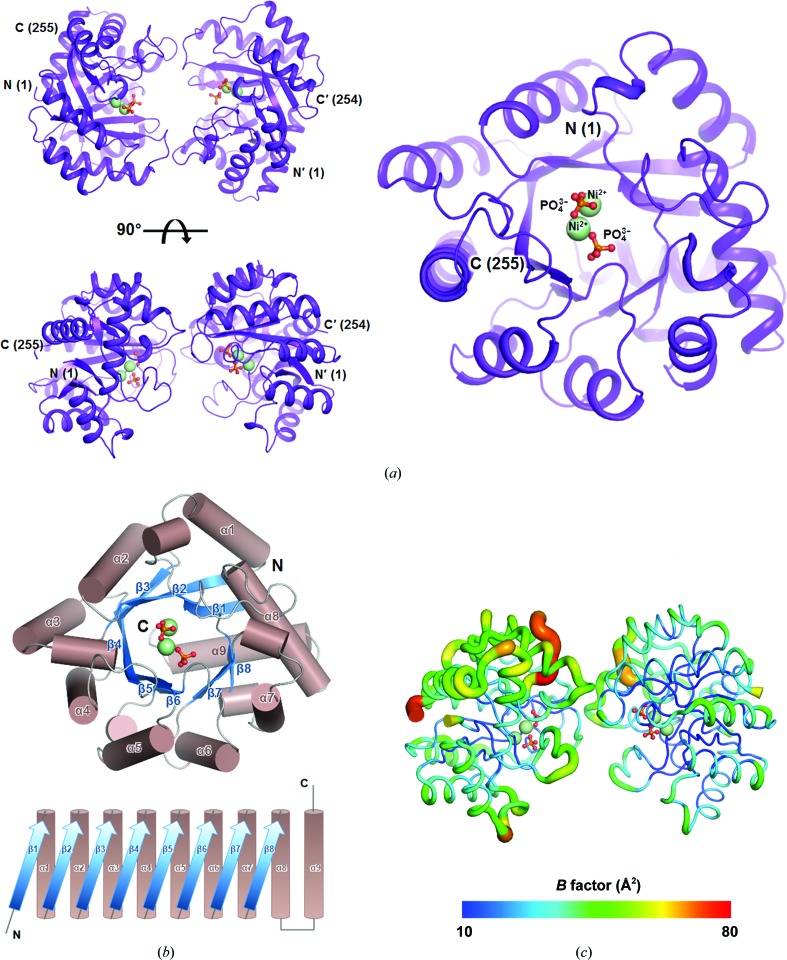
Overall structure of *Sa*TatD. (*a*) The dimeric structure of *Sa*TatD is presented as a ribbon diagram and colored purple. The Ni^2+^ ions are shown as spheres colored pale green; phosphates are shown in ball-and-stick representation with P and O atoms in orange and red, respectively. (*b*) The topology of the TIM-barrel fold constituting the *Sa*TatD structure. Helices (brown) and strands (marine) are shown as cylindrical and ribbon diagrams, respectively. (*c*) *B*-factor distributions of *Sa*TatD. The *B*-factor range (Å^2^) of the structure is shown as a gradient colored from deep blue to red. All of the structures were constructed using *PyMOL* (version 1.8; Schrödinger).

In this study, we determined the X-ray crystal structure of *Sa*TatD, including the full-length amino-acid sequence (1–257), using SAD [Fig. 1 ▸(*a*)]. The *Sa*TatD structure was refined to *R*_work_ and *R*_free_ values of 21.1% and 23.6%, respectively, at 1.85 Å resolution. The structure of *Sa*TatD consists of two monomers containing 509 amino-acid residues, four nickel ions (Ni^2+^), four phosphate ions (PO_4_^3−^) and 171 water molecules in the asymmetric unit [Fig. 1 ▸(*a*) and Table 1 ▸]. A monomer of *Sa*TatD comprises nine α-helices and eight β-strands as follows: β1 (residues 2–7), α1 (residues 18–28), β2 (residues 30–37), α2 (residues 40–52), β3 (residues 56–60), α3 (residues 72–82), β4 (residues 87–96), α4 (residues 104–121), β5 (residues 125–130), α5 (residues 133–142), β6 (residues 150–152), α6 (residues 159–167), β7 (residues 172–175), α7 (residues 185–193), β8 (residues 199–201), α8 (residues 220–234) and α9 (residues 238–252) [Fig. 1 ▸(*b*)]. In this model, most of the residues of *Sa*TatD were clearly observed, but several regions were also disordered: (i) the N-terminal residues including the hexahistidine tag (6×His), linker (Ser-Ser-Gly) and thrombin cleavage site (Leu-Val-Pro-Arg-Gly-Ser) in both chains *A* and *B* of *Sa*TatD and (ii) the C-terminal residues (Asn256–Ser257 in chain *A* and Leu255–Ser257 in chain *B* of *Sa*TatD). Two monomers of *Sa*TatD in the asymmetric unit were observed with a root-mean-square (r.m.s.) deviation of 0.19 Å for 254 equivalent C^α^-atom pairs, indicating that the structures of the two monomers are highly similar to each other.

**Figure 2:**
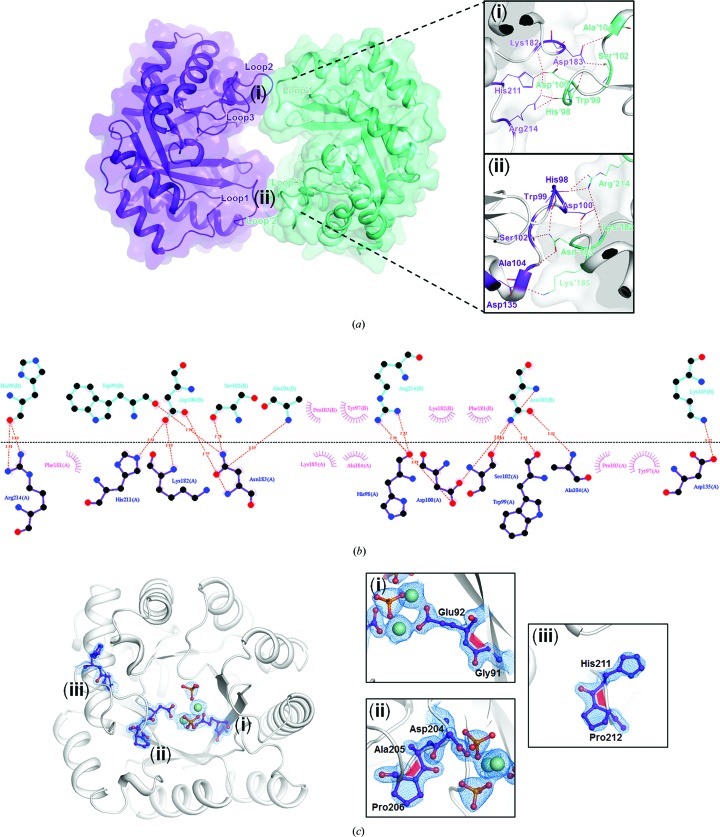
Distinct features of the *Sa*TatD structure. (*a*) Chains *A* and *B* are shown as ribbon diagrams within the transparent surface colored purple and cyan, respectively. The dimeric interface areas consisting of Loop′1–Loop2–Loop3 (i) and Loop1–Loop′2–Loop′3 (ii) are expanded in the right panel. The residues contributing to hydrogen-bonding interactions are labeled and shown as lines. The interactions are presented as dotted lines (red). (*b*) *LigPlot* diagram presenting hydrophilic (red dotted lines) and hydrophobic (pink spoked curves) interactions in the dimeric interface. (*c*) The *cis*-peptide bonds observed in the *Sa*TatD structure. Each *cis*-peptide is enlarged in the right panel (i, ii and iii) and shown as a red trapezoid. The related residues are shown in ball-and-stick representation with 2*mF*_o_ − *DF*_c_ electron-density maps contoured at 2.0σ as a blue mesh.

The overall structure of *Sa*TatD showed a TIM-barrel fold (Brändén, 1991 ▸; Nagano *et al.*, 2002 ▸), which is one of the most common folds in metabolizing enzymes, with eight β/α pairs folded into a barrel-shaped structure including an additional α-helix [Fig. 1 ▸(*b*)]. In this fold, an inner eight-stranded parallel β-sheet was surrounded by nine outer α-helices, and the active site is located in the center of the barrel structure [Fig. 1 ▸(*b*)]. The overall *B* factors were higher for the *A* chain (28 Å^2^) than for the *B* chain (23 Å^2^) in the structure of *Sa*TatD, and the highest *B* factors (52 Å^2^) were observed in the loop composed of residues 156–160 in the *A* chain, suggesting flexibility in this region (Fig. 1 ▸ *c*). The dimeric interfaces in *Sa*TatD were composed of the pairs Loop2 (Loop′2) between β7 and α7 (β′7 and α′7), Loop3 (Loop′3) between β8 and α8 (β′8 and α′8), and Loop′1 (Loop1) between β′4 and α′4 (β4 and α4) [Fig. 2 ▸(*a*)]. In this interface area, His′98, Trp′99, Asp′100, Ser′102 and Ala′104 in Loop′1 formed hydrogen bonds to Lys182 and Asn183 in Loop2 and to His211 and Arg214 in Loop3. The interactions on the opposite side of the homodimer interfaces were nearly identical. The hydrophobic interactions contributing to dimeric interfaces are represented in Fig. 2 ▸(*b*) together with the hydrophilic interactions. The buried surface area between monomers was calculated to be 580 Å^2^, and the complex-formation significance score (CSS) was determined to be 0.000 using the *PISA* server (Krissinel & Henrick, 2007 ▸), indicating that the interface area has little relevance to the formation of the dimeric structure. To identify the assembly of *Sa*TatD in solution, we performed size-exclusion chromatography using a gel-filtration standard. The result showed that low and high concentrations of *Sa*TatD protein formed monomeric and dimeric structures, respectively, in solution (Supplementary Fig. S1). This suggests that a high concentration of the protein in crystallization resulted in an artificial dimeric form of our crystal structure, and *Sa*TatD is present as a monomeric form in the cellular environment.

In this structure, unusual *cis*-peptide bonds were also observed at Gly91–Glu92, Ala205–Pro206 and His211–Pro212. They were located in the active site (Gly91–Glu92 and Ala205–Pro206) and dimeric interface (His211–Pro212), with clear electron-density maps [Fig. 2 ▸(*c*)]. Since *cis*-peptide bonds are mostly found to occur at sites critical to biological function (Williams *et al.*, 2018 ▸), these three *cis*-peptide bonds are expected to play a critical role in the catalytic mechanism of *Sa*TatD.

### 3.2. Structural comparisons of *Sa*TatD with its homologs   

**Figure 3:**
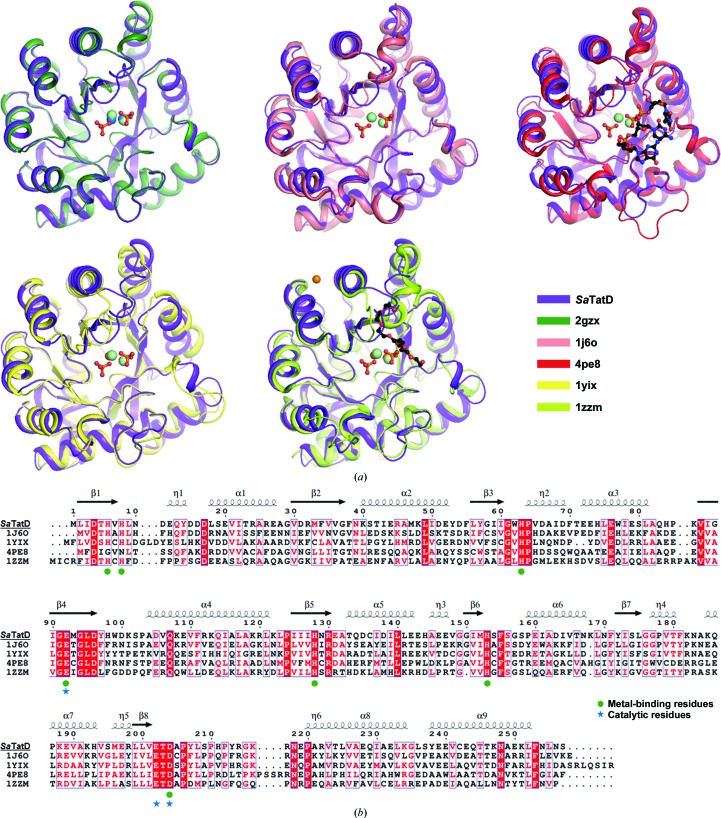
Structural comparisons of *Sa*TatD and its homologs. (*a*) Superimposed views of the monomers of *Sa*TatD and its homologs identified by the *DALI* server: TatD-like DNase from *S. aureus* (strain MW2) (green; PDB entry 2gzx), TatD-like DNase from *T. maritima* (salmon; PDB entry 1j6o), the TatD–DNA complex from *E. coli* (red; PDB entry 4pe8), YcfH from *E. coli* (yellow; PDB entry 1yix) and YjjV from *E. coli* (lime; PDB entry 1zzm). All metals and ligands are shown as spheres and in ball-and-stick representation, respectively. The Ni^2+^ ion in PDB entry 2gzx and the Zn^2+^ ion in YcfH and YjjV are colored marine and orange, respectively. The C atoms of DNA (in PDB entry 4pe8) and the PEG ligand (in PDB entry 1zzm) are presented in ball-and-stick representation in black. (*b*) Multiple sequence alignment of homologs was performed using *ClustalW* and *ESPript*. The conserved residues and similar residues are presented in white on a red background and in red in blue boxes, respectively. The residues contributing to metal binding and catalysis are marked with green circles and blue stars, respectively.

A structural similarity search using the *DALI* server (Holm & Rosenström, 2010 ▸) revealed homologs from the PDB that are similar to the monomer structure of *Sa*TatD. The top five similar structures that were identified are the following TatD-related deoxyribonucleases: (i) a TatD-like deoxyribonuclease from *S. aureus* (strain MW2) (PDB entry 2gzx; *Z*-scores of 47.1–47.2, r.m.s. deviations of 0.3–0.4 Å, sequence identity of 100% for 253 equivalent C^α^ pairs; Northeast Structural Genomics Consortium, unpublished work), (ii) a TatD-like deoxyribonuclease from *Thermotoga maritima* (PDB entry 1j6o; *Z*-score of 39.8, r.m.s. deviation of 1.3 Å, sequence identity of 39% for 254 equivalent C^α^ pairs; Joint Center for Structural Genomics, unpublished work), (iii) a TatD–DNA complex from *E. coli* (PDB code 4pe8; *Z*-scores of 38.5–38.6, r.m.s. deviation of 1.3 Å, sequence identity of 30% for 251 equivalent C^α^ pairs; Chen, Li *et al.*, 2014 ▸), (iv) YcfH from *E. coli* (PDB entry 1yix; *Z*-scores of 37.9–38.0, r.m.s. deviations of 1.5 Å, sequence identity of 38% for 251 equivalent C^α^ pairs; New York SGX Research Center for Structural Genomics, unpublished work) and (v) YjjV from *E. coli* (PDB entry 1zzm; *Z*-score of 38.0, r.m.s. deviation of 1.6 Å, sequence identity of 29% for 253 equivalent C^α^ pairs; New York SGX Research Center for Structural Genomics, unpublished work) [Fig. 3 ▸(*a*)].

All of the crystal structures of the homologs from various species described above have been deposited in the PDB without associated publications except for *E. coli* TatD (PDB entry 4pe8; Chen, Li *et al.*, 2014 ▸). All of these TatD homologs share a similar TIM-barrel fold, and several of them coordinate metal ions [Fig. 3 ▸(*a*)]. The structure of a TatD-like DNase from strain MW2 of *S. aureus* (PDB entry 2gzx) shares 100% sequence identity with our structure from strain Mu50 of *S. aureus*. However, our structural data were collected and exclusively determined using SAD at a resolution of 1.85 Å, which was higher than the 2.2 Å resolution of PDB entry 2gzx, and the refinement status was also much better than that of PDB entry 2gzx. Moreover, we confirmed that the active site in our structure clearly coordinated two Ni^2+^ ions with two phosphates, while PDB entry 2gzx showed the coordination of only two Ni^2+^ ions [Fig. 3 ▸(*a*)]. A multiple sequence alignment of homologs was performed using *ClustalW* (Chenna *et al.*, 2003 ▸) and *ESPript* (Gouet *et al.*, 1999 ▸) and is shown in Fig. 3 ▸(*b*). The alignments showed conserved residues in all homologs: we determined that His6, His8, His63, Glu92, His128, His153 and Asp204 may contribute to the binding of metal ions, and that Glu92, Glu202 and Asp204 may play a crucial role in catalytic reactions.

### 3.3. Active site of *Sa*TatD   

**Figure 4:**
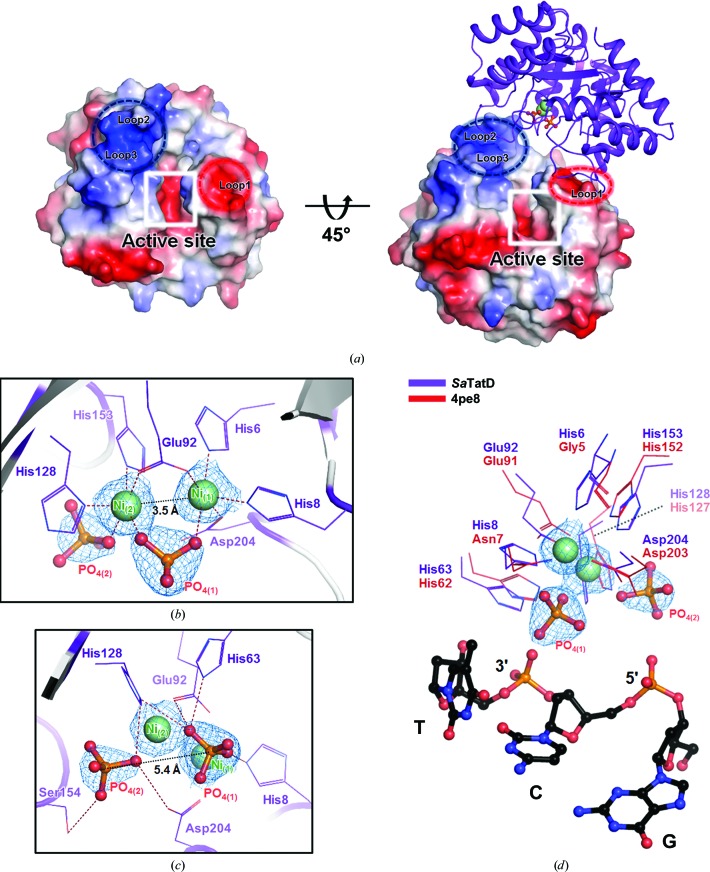
The active site of *Sa*TatD. (*a*) Electrostatic surface potential view of *Sa*TatD. The acidic active-site pocket is marked with a white box, and basic (blue) and acidic (red) surfaces composed of Loop2–Loop3 and Loop1, respectively, are marked in dotted circles. (*b*) Metal-binding site of *Sa*TatD. The key residues and two phosphates coordinating Ni^2+^ ions are labeled and shown as lines and in ball-and-stick representation, respectively. The interactions are shown as red dotted lines. The distance between the Ni^2+^ ions is labeled and is shown as a black dotted line. (*c*) The key residues contributing to the binding of phosphates are labeled and shown as lines. The distance between the P atoms of the phosphates is labeled and is shown as a black dotted line. (*d*) Superimposed view of *Sa*TatD and the *E. coli* TatD–DNA complex (PDB entry 4pe8), revealing that the two phosphates of *Sa*TatD resemble the scissile phosphates of DNA in *E. coli* TatD. T, thymine; C, cytosine; G, guanine. In (*b*–*d*), 2*mF*_o_ − *DF*_c_ electron-density maps contoured at 2.0σ are shown as a blue mesh.

Although TatD-related homologs have a highly similar fold, their active sites either bind diverse metal ions or contain no metal ions [Fig. 3 ▸(*a*)]. In this study, the clear electron density of the *Sa*TatD structure at 1.85 Å resolution provided a unique opportunity to understand the metal-binding site of TatD. An electrostatic surface potential view of *Sa*TatD showed an acidic pocket in the center of eight-stranded parallel β-sheets with basic and acidic surface areas on both sides of the pocket [Fig. 4 ▸(*a*)]. The acidic pocket is the metal-binding site, and the basic and acidic surface areas contribute to the dimeric interface. Regarding the metal-binding site, apparent electron density for two metal ions was observed. ICP-MS analysis using purified *Sa*TatD or *Sa*TatD crystals determined that these two metal-binding sites were occupied by nickel(II) (Ni^2+^) ions (Supplementary Table S2). Consequently, four Ni^2+^ ions were observed in the asymmetric unit, with two of the Ni^2+^ ions coordinated to each monomer [Fig. 4 ▸(*b*)]. The two Ni^2+^ ions in a monomer were located at the acidic pocket and towards the solvent channel. Two phosphates and the following residues coordinated the metal ions: His6, His8, His63, Glu92, His128, His153 and Asp204 [Fig. 4 ▸(*b*)]. Ni_(1)_ interacted with the side chains of His6, His8, Glu92 and Asp204 and with PO_4(1)_. Ni_(2)_ was coordinated by the side chains of Glu92, His128 and His153 and by PO_4(1)_ and PO_4(2)_. The distance between the two Ni^2+^ ions was 3.5 Å, and the O atoms of Glu92 and PO_4(1)_ formed bridges between the two Ni^2+^ ions [Fig. 4 ▸(*b*)]. Polar interactions were also observed between the two phosphates and the surface residues of *Sa*TatD [Fig. 4 ▸(*c*)]. PO_4(1)_ and PO_4(2)_ formed polar interactions with His8, His63, Glu92 and His128, and with His128, Ser154 and Asp204, respectively, where the distance between the two phosphates was 5.4 Å [Fig. 4 ▸(*c*)]. We identified that the two phosphates [PO_4(1)_ and PO_4(2)_] in the active site resemble the scissile phosphates (3′- and 5′-phosphates) of DNA in the structure of *E. coli* TatD (PDB entry 4pe8) [Fig. 4 ▸(*d*)], suggesting that metal- and phosphate-binding residues may play a key role in both metal binding and DNA hydrolysis.

### 3.4. *Sa*TatD employs Mg^2+^ ions for nuclease activity   

**Figure 5:**
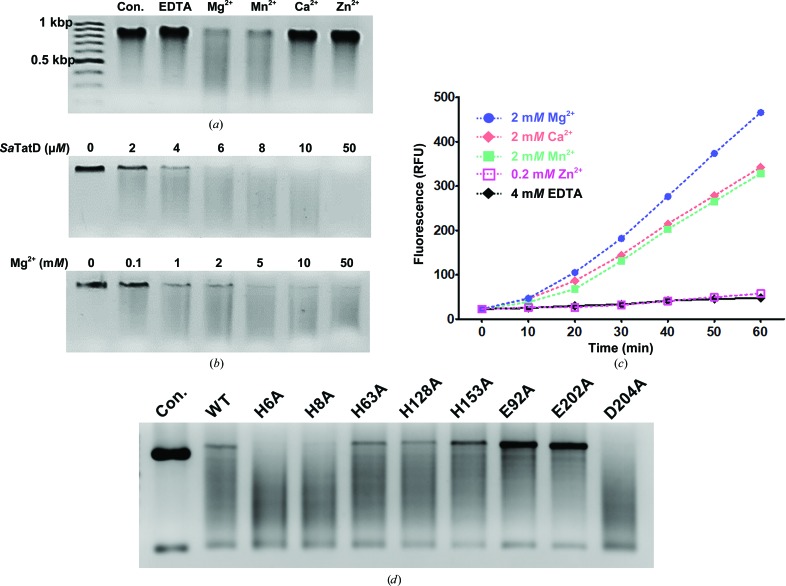
Nuclease activity assay of *Sa*TatD. (*a*) A metal-dependent DNase assay performed with 10 µ*M* protein and 4 m*M* EDTA or 2 m*M* bivalent metals with 200 n*M* *Sa*TatD genomic DNA (771 bp) as a substrate for 1 h at 37°C. Control groups (Con.) were prepared without *Sa*TatD protein. (*b*) Protein or metal concentration-dependent DNase assay carried out with 2 m*M* Mg^2+^ or 4 µ*M* protein, respectively, in the presence of 100 n*M* substrate for 1 h at 37°C. (*c*) Metal-dependent RNase assay. Fluorescence values (relative fluorescence units; RFU) were measured at Ex/Em = 490/520 nm for 1 h at 37°C. (*d*) DNase activity assay for the mutational study of *Sa*TatD carried out with each mutant protein at 4 µ*M*, 2 m*M* Mg^2+^, 200 n*M* *Sa*TatD genomic DNA for 1 h at 37°C.

To identify the metal ions that are favorable for catalytic reactions, a metal-dependent nuclease assay was performed with 2 m*M* Mg^2+^, Mn^2+^ and Ca^2+^ and 0.2 m*M* Zn^2+^ ions using full-sequence genomic DNA of *Sa*TatD as a DNA substrate. Considering previous work on *E. coli* TatD (Chen, Li *et al.*, 2014 ▸) and research which showed that a high concentration of Zn^2+^ can inhibit apoptosis, DNA fragmentation and nuclease activity (Giannakis *et al.*, 1991 ▸; Ku *et al.*, 2002 ▸), we adopted a tenfold lower concentration of Zn^2+^ than the other metal ions for the nuclease assay. In the DNase assay, a control group without *Sa*TatD protein and an EDTA-treated group showed a similar band width, indicating that *Sa*TatD without metal ions did not perform DNA hydrolysis [Fig. 5 ▸(*a*)]. Both the Mg^2+^- and Mn^2+^-treated groups revealed explicit DNase activity, but the Mg^2+^-treated group showed slightly stronger activity than the Mn^2+^-treated group. In contrast, the Ca^2+^- and Zn^2+^-treated groups showed similar DNase activity to that of the control and EDTA-treated groups. Consistently, *Sa*TatD presented protein and metal (Mg^2+^) concentration-dependent DNase activity [Fig. 5 ▸(*b*)]. Furthermore, we performed a metal-dependent RNase assay, which showed a different tendency from the DNase assay. *Sa*TatD exhibited relative activity levels in the presence of different metals as follows: Mg^2+^ > Ca^2+^ > Mn^2+^ >> Zn^2+^ = EDTA [Fig. 5 ▸(*c*)]. Taken together, these results confirmed that *Sa*TatD bears Mg^2+^-dependent nuclease activity as a DNase and an RNase.

### 3.5. Glu92 and Glu202 have a crucial effect on the catalytic activity of *Sa*TatD   

A comparative analysis of the active sites of *Sa*TatD and its homologs provided considerable insight into the catalytic mechanism of *Sa*TatD. To further verify the key residues involved in DNA hydrolysis by *Sa*TatD, we performed eight single-point mutations of the residues in the active site, H6A, H8A, H63A, E92A, H128A, H153A, E202A and D204A, as presented in Supplementary Table S1. We confirmed that the alanine mutants of the histidine cluster, including H6A, H8A, H128A, H153A and H63A, did not show significant alterations in DNase activity, despite these residues being key in metal and phosphate binding [Fig. 5 ▸(*d*)]. Asp204 was expected to contribute to the catalytic reaction, similar to its counterpart in *E. coli* TatD (Chen, Li *et al.*, 2014 ▸), but its alanine mutant showed no meaningful alteration of the activity [Fig. 5 ▸(*d*)]. On the other hand, the mutants of the conserved residues Glu92 and Glu202 revealed an apparent reduction in DNase activity, indicating that these two glutamates are key residues for the catalytic reaction of *Sa*TatD.

### 3.6. Putative DNA-binding mode of *Sa*TatD   

**Figure 6:**
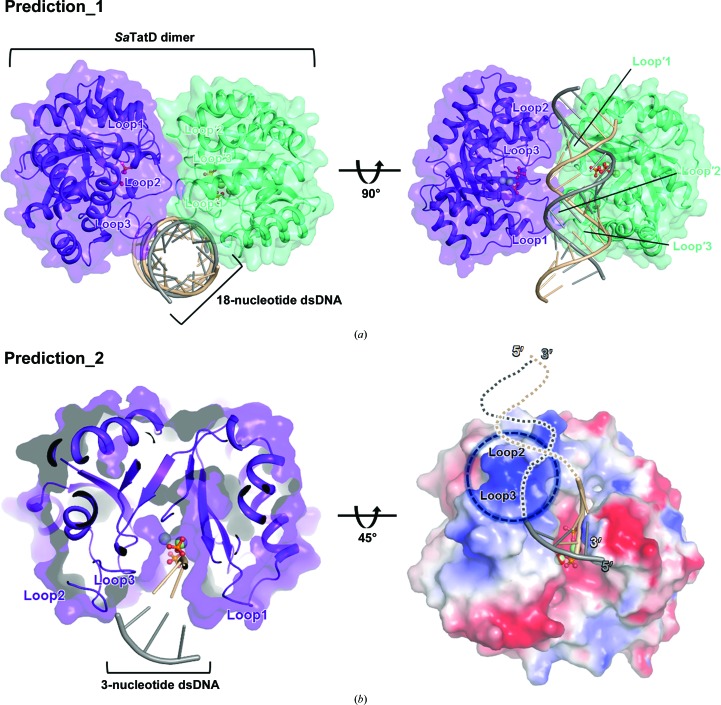
Proposed DNA-binding mode. (*a*) Prediction_1 for the DNA-binding mode of the *Sa*TatD dimer with 18-mer dsDNA. Prediction_1 suggests that owing to the dimeric interface, DNA has difficulty approaching the active site of *Sa*TatD. DNA is shown as a ribbon diagram, and the DNA strands are colored wheat (3′–5′) and dark gray (5′–3′). (*b*) Prediction_2 for the DNA-binding mode of the *Sa*TatD monomer with 3-mer dsDNA considering structural hindrance. Elongated DNA chains that may bind to the basic surface area (blue dotted circle) consisting of Loop2–Loop3 are displayed as wheat and dark gray dotted lines.

In this study, we observed clear electron density for two phosphates in the crystal structure of *Sa*TatD. As mentioned above, a comparative analysis with the TatD–DNA complex structure from *E. coli* (PDB entry 4pe8) revealed that the binding positions of these two phosphates considerably resemble those of the scissile phosphates of DNA in *E. coli* TatD [Fig. 4 ▸(*d*)]. However, the two phosphates in our structure were closely bound to two Ni^2+^ ions, whereas PDB entry 4pe8 revealed a loose binding mode for the cleaved DNA, depicting the inactive DNA-bound form. To further understand the active DNA-binding mode of *Sa*TatD, we performed *in silico* docking using *HADDOCK* (Dominguez *et al.*, 2003 ▸) with His8, His63, Glu92, His128, Ser154 and Asp204 selected as ‘active residues’ based on the binding mode of the phosphates. We hypothesized that *Sa*TatD would bind to double-stranded DNA (dsDNA) in the dimeric structure [Fig. 6 ▸(*a*)]. However, Prediction_1 [Fig. 6 ▸(*a*)] proposed that dsDNA could not enter the active site in the dimeric structure owing to Loop1, Loop2 and Loop3, which were on both sides of the active site and formed the dimeric interface of *Sa*TatD [Figs. 2 ▸(*a*) and 4 ▸(*a*)]. Considering the structural hindrance, we predicted the binding mode of the *Sa*TatD monomer and a 3-mer dsDNA [Fig. 6 ▸(*b*)]. Prediction_2 [Fig. 6 ▸(*b*)] proposed that the 3-mer dsDNA might form interactions between the two interface areas of the *Sa*TatD monomer composed of Loop1 or Loop2 and Loop3, revealing a binding mode similar to that of the two phosphates of *Sa*TatD. Furthermore, an electrostatic view showed that the basic area composed of Loop2 and Loop3 on the left-side top region may contribute to the binding of elongated DNA [Fig. 6 ▸(*b*)]. The DNA-binding mode of the *Sa*TatD monomer agrees with the SEC results using the gel-filtration standard. Taken together, the results of the structural and mutational studies and our docking model suggest a DNA-binding mode for monomeric *Sa*TatD.

## 4. Discussion   

Despite interest in the Tat system that exports completely folded proteins, TatD has not thoroughly been investigated since there was no direct functional relationship between TatD and the other Tat proteins, including TatA, TatB and TatC. However, because TatD is an evolutionarily conserved protein and encoded in the *tat* operon with other Tat proteins (Sargent *et al.*, 1998 ▸), this protein must play a crucial cellular role. Indeed, TatD has been reported to be relevant to the Tat-linked quality-control system, the degradation of abnormal proteins, apoptosis and DNA repair (Matos *et al.*, 2009 ▸; Chen, Li *et al.*, 2014 ▸; Qiu *et al.*, 2005 ▸; Chen, Shen *et al.*, 2014 ▸). In this study, our in-depth insights into *Sa*TatD, a TatD-related nuclease from *S. aureus*, revealed the unique structural and functional features of the TatD proteins from Gram-positive bacteria.

This study describes the crystal structure of *Sa*TatD at the atomic level using the SAD method. The previous Ni^2+^-bound monomeric structure (PDB entry 2gzx) from *S. aureus*, which shares 100% sequence identity with *Sa*TatD, gave 2.2 Å resolution data that were less refined and were deposited in the PDB without an associated publication. In contrast, our dimeric structure of *Sa*TatD is a clear atomic description at high resolution (1.85 Å) with unique binding of Ni^2+^ ions and phosphates. Although the overall structure of *Sa*TatD had the ubiquitous TIM-barrel fold (Brändén, 1991 ▸; Nagano *et al.*, 2002 ▸), the active site showed an intriguing binding mode of ligands that differed from other TatD homologs. To the best of our knowledge, there have not been reports of TatD structures with coordination by two phosphates. The two phosphates resembled considerably the scissile phosphates of three-nucleotide single-stranded DNA complexed with TatD from *E. coli* (PDB entry 4pe8; Chen, Li *et al.*, 2014 ▸), and thus we concluded that the residues contributing to the binding of Ni^2+^ ions and phosphates could play a key role in the DNase activity of *Sa*TatD. Moreover, we confirmed one nonproline and two proline *cis*-peptide bonds in this structure. A majority of enzymes usually adopt the *trans* form rather than the *cis* form. The occurrence rate of *cis*-Pro is slightly over 5%, and *cis*-nonPro is extremely rare (Williams *et al.*, 2018 ▸). Thus, the presence of *cis*-peptides in this structure implies an important functional role. Indeed, Gly91–*cis*-Glu92 and Ala205–*cis*-Pro206 are likely to considerably affect the conformations of the conserved residues Glu92 and Asp204 and the active site. In His211–*cis*-Pro212, located in Loop3, His211 contributes to the conformation of the interface area, but apart from that the binding mode suggests that this basic residue may serve as a DNA-binding residue.

In contrast to the structural description of metal ions, we identified that *Sa*TatD has more efficient DNase and RNase activity with Mg^2+^ ions than with Mn^2+^ ions, which is consistent with previous TatD-related research (Lindenstrauss *et al.*, 2010 ▸; Wexler *et al.*, 2000 ▸). A mutational study to further explore the catalytic mechanism of *Sa*TatD revealed that the two residues Glu92 and Glu202 have a critical effect on catalysis by *Sa*TatD, even though the conserved residue Glu202, in particular, had no structural relevance to metal or phosphate binding. Mutants with changes at His63 and Asp204 rarely showed significant alterations in activity, although these residues play an important role in hydrolysis by *E. coli* TatD. In addition, *E. coli* TatD only showed nuclease activity for single-stranded nucleic acids, while *Sa*TatD efficiently trimmed dsDNA. This functional difference may be caused by the distinct difference in catalytic residues and may be owing to the evolutionary selection for a specific activity within the ubiquitous TIM-barrel fold (Singh *et al.*, 2019 ▸).

As mentioned above, superposition of the *Sa*TatD and *E. coli* TatD–DNA complexes revealed that the two phosphates bound to *Sa*TatD are located in a similar position to the DNA bound to *E. coli* TatD. PO_4(1)_ bound to *Sa*TatD was located near the 3′-end phosphate of the DNA in *E. coli* TatD, and PO_4(2)_ was proximal to the 5′-end phosphate of the DNA in *E. coli* TatD. In addition, considering the presence of the only basic surface area to be identified near the active site of *Sa*TatD, the 5′-end of DNA should extend to the basic area, whereas the 3′-end of DNA should enter the active site of *Sa*TatD. This result corresponds to previous work on *E. coli* TatD (Chen, Li *et al.*, 2014 ▸), suggesting that *Sa*TatD may act as a 3′–5′ exonuclease, similar to *E. coli* TatD. Furthermore, in contrast to expectations, the first prediction of dsDNA binding to the *Sa*TatD dimer revealed that the dimeric interface could be an obstacle to DNA entering the active site of *Sa*TatD. Thus, we postulated that *Sa*TatD may act as a monomer for DNA hydrolysis similar to most TatD-related DNases, which was supported by the gel-filtration results. However, we could not predict the long DNA-bound structure. Instead, to minimize the structural hindrance, we constructed a three-nucleotide dsDNA for docking with a monomer of *Sa*TatD. Indeed, our second prediction proposed that the putative DNA-binding model fits in the active site well, suggesting that the basic surface area composed of Loop2 and Loop3 in the *Sa*TatD monomer may play a critical role in recognition and binding of DNA in the active site of *Sa*TatD, which may seize and trim the 3′-end of a 3′–5′ strand of the DNA duplex as a 3′–5′ exonuclease.

In conclusion, we have unveiled the crystal structure of *Sa*TatD, a TatD-related nuclease from the Gram-positive organism *S. aureus*, and have provided the first description of a unique binding mode of Ni^2+^ ions and phosphates in TatD homologs. Structural insights into the active site of *Sa*TatD and a mutational study provided further understanding of its catalytic mechanism. Furthermore, based on the unique binding mode of two phosphates, we suggested a DNA-binding mode for active *Sa*TatD monomers. Our findings provide considerable insights into the relationships between the structure and mechanism of TatD proteins from Gram-positive bacteria and will be a valuable basis for research in fields related to TatD.

## Supplementary Material

Supporting information. DOI: 10.1107/S2052252520003917/lz5034sup1.pdf

PDB reference: TatD, 6l25

## Figures and Tables

**Table 1:** Data-collection and model-refinement statistics for SeMet-derived *Sa*TatD (PDB entry 6l25) Values in parentheses are for the highest resolution shell.

Data collection	
X-ray wavelength (Å)	0.9793
Space group	*P*2_1_
*a*, *b*, *c* (Å)	47.62, 77.83, 77.38
α, β, γ (°)	90.00, 98.81, 90.00
Resolution range (Å)	30.00–1.85 (1.88–1.85)
Total/unique reflections	452923/47022 (2125)
Completeness (%)	98.6 (89.4)
CC_1/2_ †	0.989 (0.872)
〈*I*/σ(*I*)〉	41.9 (4.1)
*R*_merge_ ‡	0.112 (0.760)
Model refinement	
*R*_work_/*R*_free_ §	0.210/0.236
No. of atoms	
Protein atoms	4088
Water O atoms	171
Ligand atoms	
Nickel(II) (Ni^2+^)	4
Phosphate (PO_4_^3−^)	4
Average *B* factor (Å^2^)	
Protein atoms	27.29
Water O atoms	30.27
Ligand atoms	
Nickel(II) (Ni^2+^)	22.48
Phosphate (PO_4_^3−^)	34.38
R.m.s. deviations from ideal geometry	
Bond lengths (Å)	0.012
Bond angles (°)	1.769
Ramachandran plot (%)	
Most favorable	97.03
Allowed	2.97
Disallowed	0.00

† CC_1/2_ is described in Karplus & Diederichs (2012 ▸).

‡ *R*_merge_ = 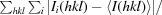 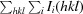, where *I*(*hkl*) is the intensity of reflection *hkl*, 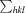 is the sum over all reflections and 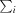 is the sum over *i* measurements of reflection *hkl*.

§ *R* = 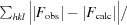 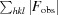, where *R*_free_ is calculated for a randomly chosen 5% of reflections which were not used for structure refinement and *R*_work_ is calculated for the remaining reflections.
