# Supplementary material for: A structural study of TatD from Staphylococcus aureus elucidates a putative DNA-binding mode of a Mg2+-dependent nuclease
Source: IUCrJ. 2020 Apr 17;7(Pt 3):509–21. doi: 10.1107/S2052252520003917 (PMC7201278; doi:10.1107/S2052252520003917)
Supplement: Supplementary file 1 [file m-07-00509-sup1.pdf]

# IUCrJ

**Volume 7 (2020)**


**Supporting information for article:**

## A structural study of TatD from *Staphylococcus aureus* elucidates a putative DNA-binding mode of a $Mg^{2+}$-dependent nuclease

**Kyu-Yeon Lee, Seung-Ho Cheon, Dong-Gyun Kim, Sang Jae Lee and Bong-Jin Lee**

**Supporting information**

**Table S1** Primer design for SAV0491 wild type and mutants. Sites for site-directed mutagenesis are underlined in black. Single-letter abbreviations for amino acid residues: H, histidine; A, alanine; E, glutamic acid; and D, aspartic acid.

| Mutated sites | | Primer sequence (5'→3') |
|---|---|---|
| H6A | Forward | G TTA ATC GAT ACA GCC GTC CAT TTA AAT GAT G |
| | Reverse | C ATC ATT TAA ATG GAC GGC TGT ATC GAT TAA C |
| H8A | Forward | C GAT ACA CAT GTC GCC TTA AAT GAT GAG CAA TAC |
| | Reverse | GTA TTG CTC ATC ATT TAA GGC GAC ATG TGT ATC G |
| H63A | Forward | GGC ATT ATC GGT TGG GCC CCA GTT GAC GCA ATT G |
| | Reverse | C AAT TGC GTC AAC TGG GGC CCA ACC GAT AAT GCC |
| H128A | Forward | G TTA CCA ATT ATC ATT GCC AAC CGT GAA GCA ACT C |
| | Reverse | G AGT TGC TTC ACG GTT GGC AAT GAT AAT TGG TAA C |
| H153A | Forward | GTA GGC GGG ATT ATG GCC AGC TTT AGT GGT TCT C |
| | Reverse | G AGA ACC ACT AAA GCT GGC CAT AAT CCC GCC TAC |
| E92A | Forward | GTG ATT GGT ATT GGT GCC ATG GGA TTA GAT TAT C |
| | Reverse | G ATA ATC TAA TCC CAT GGC ACC AAT ACC AAT CAC |
| E202A | Forward | GAG CGT TTG CTA GTT GCC ACC GAT GCA CCG TAT C |
| | Reverse | G ATA CGG TGC ATC GGT GGC AAC TAG CAA ACG CTC |
| D204A | Forward | G CTA GTT GAA ACC GCC GCA CCG TAT CTC TCG |
| | Reverse | CGA GAG ATA CGG TGC GGC GGT TTC AAC TAG C |

**Table S2** ICP-MS analysis. ND indicates “not detected”.

| | **Crystals dissolved in the water** | **SaTatD protein solution** |
|---|---|---|
| **Manganese (II)** | ND | ND |
| **Nickel (II)** | 112.000 ng/ml | 10694.000 ng/ml |
| **Zinc (II)** | ND | 4318.000 ng/ml |
| **Calcium (II)** | ND | ND |
| **Magnesium (II)** | ND | ND |

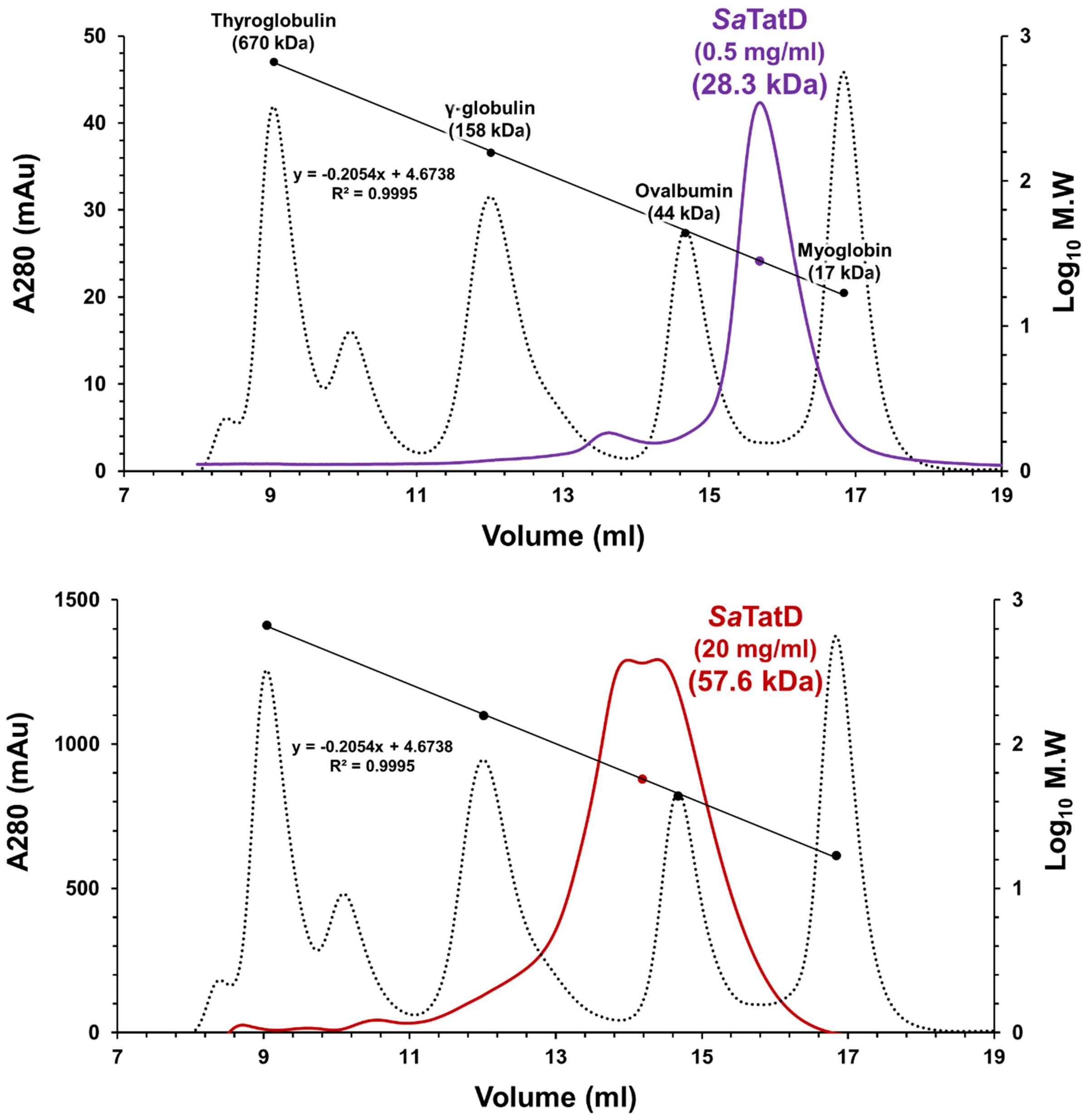


**Figure S1** Identification of the oligomeric state of *Sa*TatD in solution. Analytical gel filtration profiles of *Sa*TatD using 0.5 and 20 mg/ml proteins. The theoretical M.W. of *Sa*TatD monomer including N-terminal hexa-histidine and linker is 31.3 kDa.
